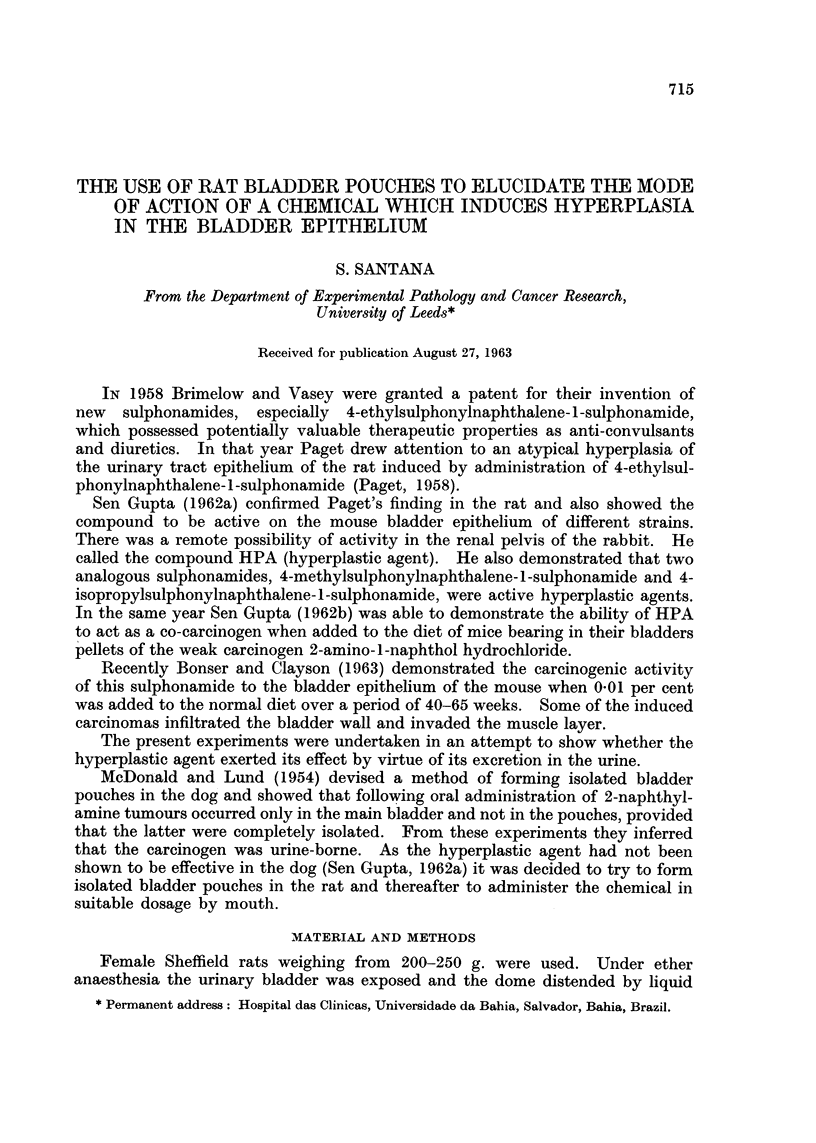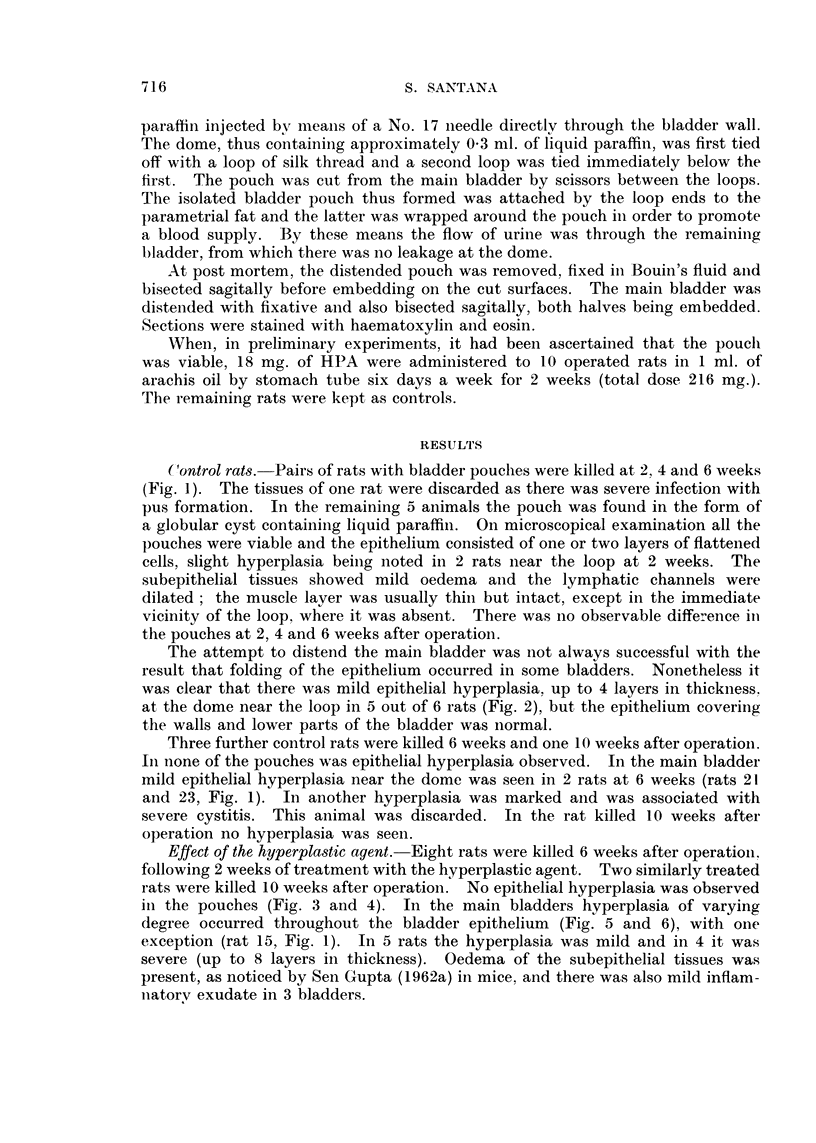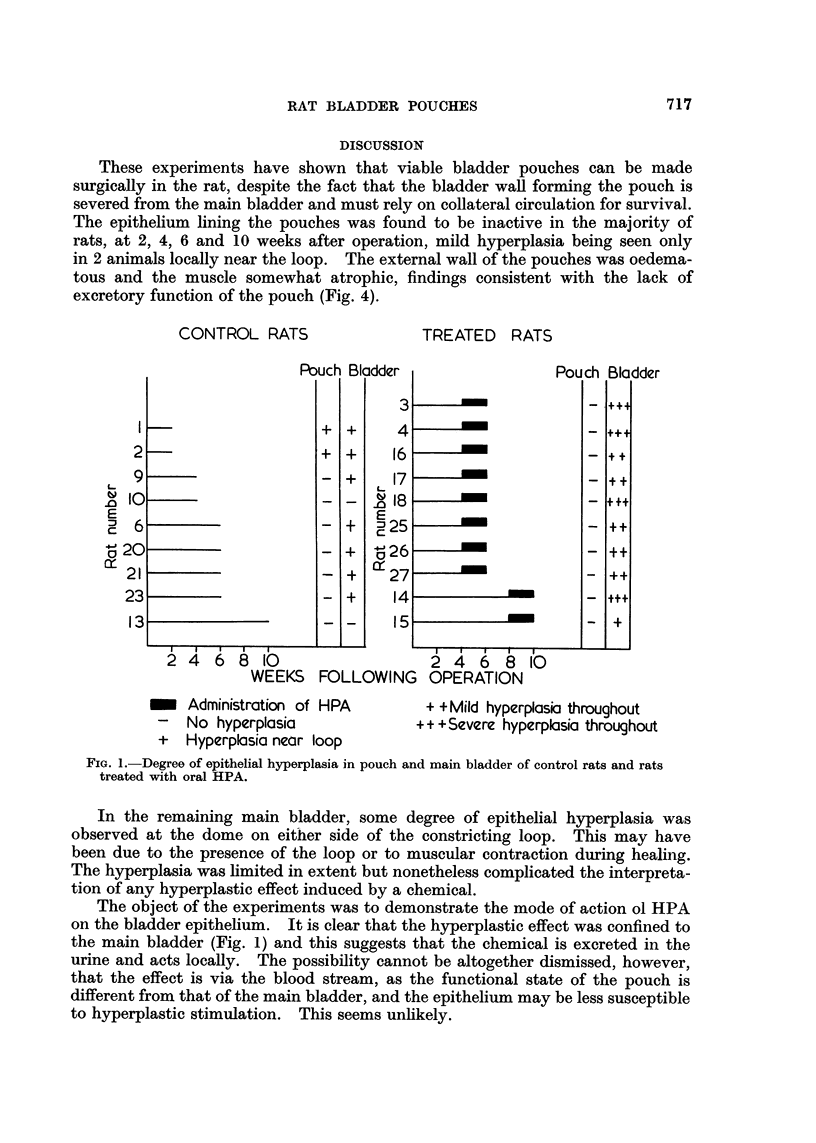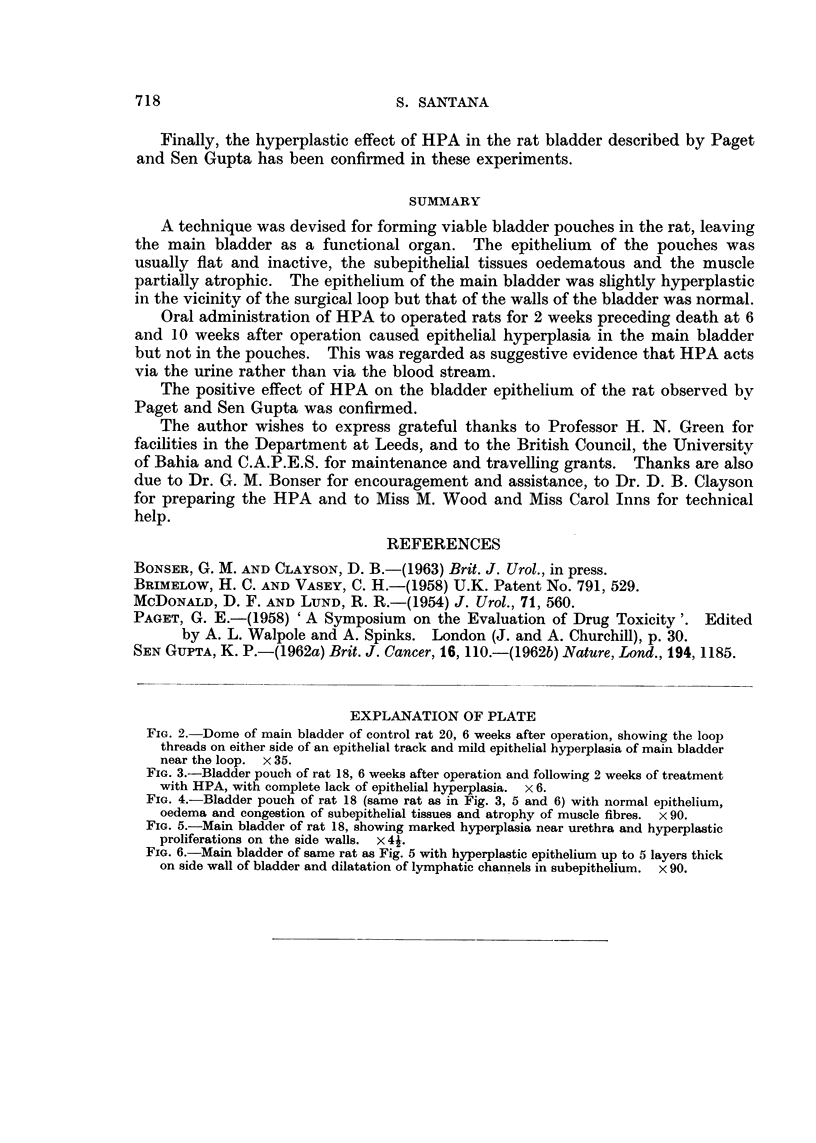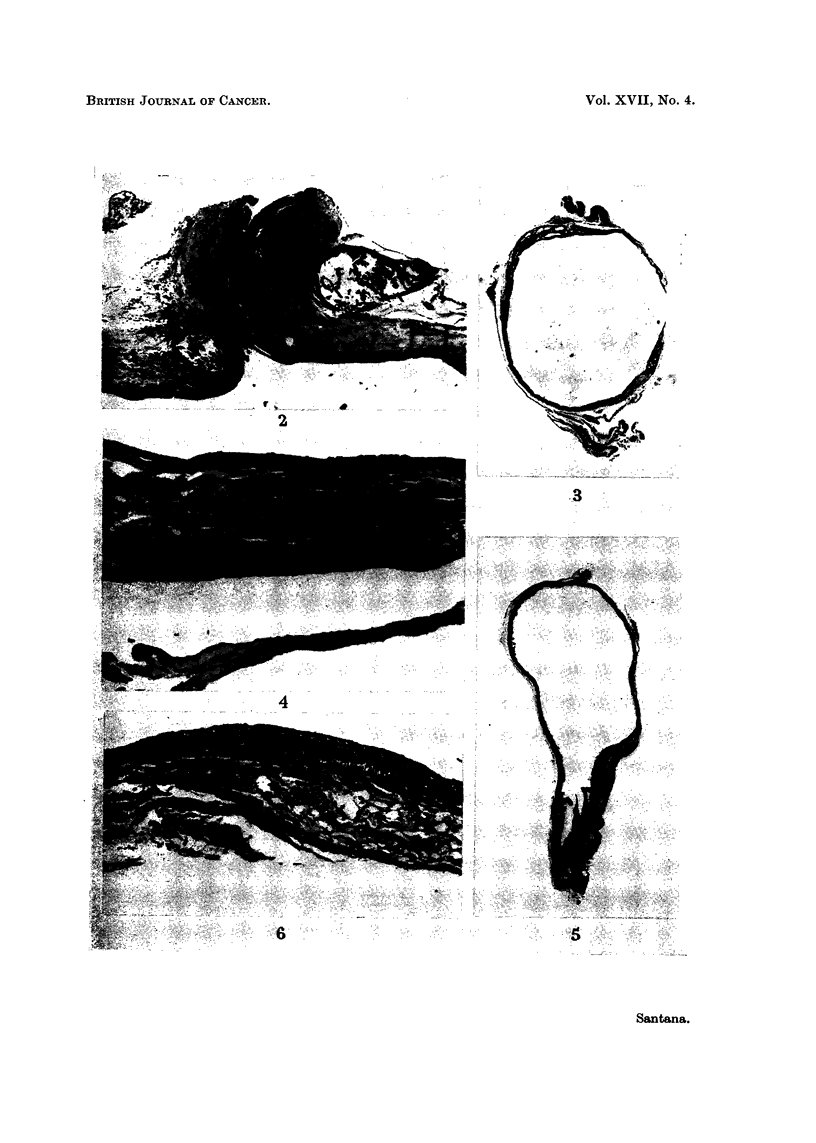# The Use of Rat Bladder Pouches to Elucidate the Mode of Action of a Chemical which Induces Hyperplasia in the Bladder Epithelium

**DOI:** 10.1038/bjc.1963.91

**Published:** 1963-12

**Authors:** S. Santana

## Abstract

**Images:**


### THE USE OF RAT BLADDER POUCHES TO ELUCIDATE THE MODE OF ACTION OF A CHEMICAL WHICH INDUCES HYPERPLASIA IN THE BLADDER EPITHELIUM

S. SANTANA

*From the Department of Experimental Pathology and Cancer Research, University of Leeds**

Received for publication August 27, 1963

IN 1958 Brimelow and Vasey were granted a patent for their invention of new sulphonamides, especially 4-ethylsulphonylnaphthalene-1-sulphonamide, which possessed potentially valuable therapeutic properties as anti-convulsants and diuretics. In that year Paget drew attention to an atypical hyperplasia of the urinary tract epithelium of the rat induced by administration of 4-ethylsulphonylnaphthalene-1-sulphonamide (Paget, 1958).

Sen Gupta (1962a) confirmed Paget's finding in the rat and also showed the compound to be active on the mouse bladder epithelium of different strains. There was a remote possibility of activity in the renal pelvis of the rabbit. He called the compound HPA (hyperplastic agent). He also demonstrated that two analogous sulphonamides, 4-methylsulphonylnaphthalene-1-sulphonamide and 4-isopropylsulphonylnaphthalene-1-sulphonamide, were active hyperplastic agents. In the same year Sen Gupta (1962b) was able to demonstrate the ability of HPA to act as a co-carcinogen when added to the diet of mice bearing in their bladders pellets of the weak carcinogen 2-amino-1-naphthol hydrochloride.

Recently Bonser and Clayson (1963) demonstrated the carcinogenic activity of this sulphonamide to the bladder epithelium of the mouse when 0·01 per cent was added to the normal diet over a period of 40–65 weeks. Some of the induced carcinomas infiltrated the bladder wall and invaded the muscle layer.

The present experiments were undertaken in an attempt to show whether the hyperplastic agent exerted its effect by virtue of its excretion in the urine.

McDonald and Lund (1954) devised a method of forming isolated bladder pouches in the dog and showed that following oral administration of 2-naphthylamine tumours occurred only in the main bladder and not in the pouches, provided that the latter were completely isolated. From these experiments they inferred that the carcinogen was urine-borne. As the hyperplastic agent had not been shown to be effective in the dog (Sen Gupta, 1962a) it was decided to try to form isolated bladder pouches in the rat and thereafter to administer the chemical in suitable dosage by mouth.

### MATERIAL AND METHODS

Female Sheffield rats weighing from 200–250 g. were used. Under ether anaesthesia the urinary bladder was exposed and the dome distended by liquid

* Permanent address : Hospital das Clinicas, Universidade da Bahia, Salvador, Bahia, Brazil.

paraffin injected by means of a No. 17 needle directly through the bladder wall. The dome, thus containing approximately 0·3 ml. of liquid paraffin, was first tied off with a loop of silk thread and a second loop was tied immediately below the first. The pouch was cut from the main bladder by scissors between the loops. The isolated bladder pouch thus formed was attached by the loop ends to the parametrial fat and the latter was wrapped around the pouch in order to promote a blood supply. By these means the flow of urine was through the remaining bladder, from which there was no leakage at the dome.

At post mortem, the distended pouch was removed, fixed in Bouin's fluid and bisected sagitally before embedding on the cut surfaces. The main bladder was distended with fixative and also bisected sagitally, both halves being embedded. Sections were stained with haematoxylin and eosin.

When, in preliminary experiments, it had been ascertained that the pouch was viable, 18 mg. of HPA were administered to 10 operated rats in 1 ml. of arachis oil by stomach tube six days a week for 2 weeks (total dose 216 mg.). The remaining rats were kept as controls.

### RESULTS

*Control rats.*—Pairs of rats with bladder pouches were killed at 2, 4 and 6 weeks (Fig. 1). The tissues of one rat were discarded as there was severe infection with pus formation. In the remaining 5 animals the pouch was found in the form of a globular cyst containing liquid paraffin. On microscopical examination all the pouches were viable and the epithelium consisted of one or two layers of flattened cells, slight hyperplasia being noted in 2 rats near the loop at 2 weeks. The subepithelial tissues showed mild oedema and the lymphatic channels were dilated ; the muscle layer was usually thin but intact, except in the immediate vicinity of the loop, where it was absent. There was no observable difference in the pouches at 2, 4 and 6 weeks after operation.

The attempt to distend the main bladder was not always successful with the result that folding of the epithelium occurred in some bladders. Nonetheless it was clear that there was mild epithelial hyperplasia, up to 4 layers in thickness, at the dome near the loop in 5 out of 6 rats (Fig. 2), but the epithelium covering the walls and lower parts of the bladder was normal.

Three further control rats were killed 6 weeks and one 10 weeks after operation. In none of the pouches was epithelial hyperplasia observed. In the main bladder mild epithelial hyperplasia near the dome was seen in 2 rats at 6 weeks (rats 21 and 23, Fig. 1). In another hyperplasia was marked and was associated with severe cystitis. This animal was discarded. In the rat killed 10 weeks after operation no hyperplasia was seen.

*Effect of the hyperplastic agent.*—Eight rats were killed 6 weeks after operation, following 2 weeks of treatment with the hyperplastic agent. Two similarly treated rats were killed 10 weeks after operation. No epithelial hyperplasia was observed in the pouches (Fig. 3 and 4). In the main bladders hyperplasia of varying degree occurred throughout the bladder epithelium (Fig. 5 and 6), with one exception (rat 15, Fig. 1). In 5 rats the hyperplasia was mild and in 4 it was severe (up to 8 layers in thickness). Oedema of the subepithelial tissues was present, as noticed by Sen Gupta (1962a) in mice, and there was also mild inflammatory exudate in 3 bladders.

### DISCUSSION

These experiments have shown that viable bladder pouches can be made surgically in the rat, despite the fact that the bladder wall forming the pouch is severed from the main bladder and must rely on collateral circulation for survival. The epithelium lining the pouches was found to be inactive in the majority of rats, at 2, 4, 6 and 10 weeks after operation, mild hyperplasia being seen only in 2 animals locally near the loop. The external wall of the pouches was oedematous and the muscle somewhat atrophic, findings consistent with the lack of excretory function of the pouch (Fig. 4).

FIG. 1.—Degree of epithelial hyperplasia in pouch and main bladder of control rats and rats treated with oral HPA.

In the remaining main bladder, some degree of epithelial hyperplasia was observed at the dome on either side of the constricting loop. This may have been due to the presence of the loop or to muscular contraction during healing. The hyperplasia was limited in extent but nonetheless complicated the interpretation of any hyperplastic effect induced by a chemical.

The object of the experiments was to demonstrate the mode of action ol HPA on the bladder epithelium. It is clear that the hyperplastic effect was confined to the main bladder (Fig. 1) and this suggests that the chemical is excreted in the urine and acts locally. The possibility cannot be altogether dismissed, however, that the effect is via the blood stream, as the functional state of the pouch is different from that of the main bladder, and the epithelium may be less susceptible to hyperplastic stimulation. This seems unlikely.

Finally, the hyperplastic effect of HPA in the rat bladder described by Paget and Sen Gupta has been confirmed in these experiments.

SUMMARY

A technique was devised for forming viable bladder pouches in the rat, leaving the main bladder as a functional organ. The epithelium of the pouches was usually flat and inactive, the subepithelial tissues oedematous and the muscle partially atrophic. The epithelium of the main bladder was slightly hyperplastic in the vicinity of the surgical loop but that of the walls of the bladder was normal.

Oral administration of HPA to operated rats for 2 weeks preceding death at 6 and 10 weeks after operation caused epithelial hyperplasia in the main bladder but not in the pouches. This was regarded as suggestive evidence that HPA acts via the urine rather than via the blood stream.

The positive effect of HPA on the bladder epithelium of the rat observed by Paget and Sen Gupta was confirmed.

The author wishes to express grateful thanks to Professor H. N. Green for facilities in the Department at Leeds, and to the British Council, the University of Bahia and C.A.P.E.S. for maintenance and travelling grants. Thanks are also due to Dr. G. M. Bonser for encouragement and assistance, to Dr. D. B. Clayson for preparing the HPA and to Miss M. Wood and Miss Carol Inns for technical help.

### REFERENCES

BONSER, G. M. AND CLAYSON, D. B.—(1963) *Brit. J. Urol.*, in press.
BRIMELOW, H. C. AND VASEY, C. H.—(1958) U.K. Patent No. 791, 529.
McDONALD, D. F. AND LUND, R. R.—(1954) *J. Urol.*, **71**, 560.
PAGET, G. E.—(1958) ' A Symposium on the Evaluation of Drug Toxicity '. Edited by A. L. Walpole and A. Spinks. London (J. and A. Churchill), p. 30.
SEN GUPTA, K. P.—(1962*a*) *Brit. J. Cancer*, **16**, 110.—(1962*b*) *Nature, Lond.*, **194**, 1185.

### EXPLANATION OF PLATE

FIG. 2.—Dome of main bladder of control rat 20, 6 weeks after operation, showing the loop threads on either side of an epithelial track and mild epithelial hyperplasia of main bladder near the loop. ×35.

FIG. 3.—Bladder pouch of rat 18, 6 weeks after operation and following 2 weeks of treatment with HPA, with complete lack of epithelial hyperplasia. ×6.

FIG. 4.—Bladder pouch of rat 18 (same rat as in Fig. 3, 5 and 6) with normal epithelium, oedema and congestion of subepithelial tissues and atrophy of muscle fibres. ×90.

FIG. 5.—Main bladder of rat 18, showing marked hyperplasia near urethra and hyperplastic proliferations on the side walls. ×4½.

FIG. 6.—Main bladder of same rat as Fig. 5 with hyperplastic epithelium up to 5 layers thick on side wall of bladder and dilatation of lymphatic channels in subepithelium. ×90.